# The Beneficial Role of the Thyroid Hormone Receptor Beta 2 (*thrb2*) in Facilitating the First Feeding and Subsequent Growth in Medaka as Fish Larval Model

**DOI:** 10.3390/cells14050386

**Published:** 2025-03-06

**Authors:** Jiaqi Wu, Ke Lu, Ruipeng Xie, Chenyuan Zhu, Qiyao Luo, Xu-Fang Liang

**Affiliations:** 1 College of Fisheries, Chinese Perch Research Center, Huazhong Agricultural University, Wuhan 430070, China; 2021308010016@webmail.hzau.edu.cn (J.W.); luke@mail.hzau.edu.cn (K.L.); xierp01321@outlook.com (R.X.); zhucy@webmail.hzau.edu.cn (C.Z.); qiyaoluo@webmail.hzau.edu.cn (Q.L.); 2 Engineering Research Center of Green development for Conventional Aquatic Biological Industry in the Yangtze River Economic Belt, Ministry of Education, Wuhan 430070, China

**Keywords:** thyroid hormone receptor beta 2, retinal development, first feeding, growth, larvae

## Abstract

During the early growth stages of fish larvae, there are significant challenges to their viability, so improving their visual environment is essential to promoting their growth and survival. Following the successful knockout of thyroid hormone receptor beta 2 (*thrb2*) using Clustered Regularly Interspaced Short Palindromic Repeats (CRISPR)/Cas9 technology, there was an increase in the expression of UV opsin (short-wave-sensitive 1, *sws1*), while the expression of other cone opsins was significantly decreased. Further analysis of the retinal structure demonstrated that the *thrb2* knockout resulted in an increased lens thickness and a decreased thickness of the ganglion cell layer (GCL), outer plexiform layer (OPL), and outer nuclear layer (ONL) in the retina. The slowing down of swimming speed under light conditions in *thrb2*^−/−^ may be related to the decreased expression of phototransduction-related genes such as G protein-coupled receptor kinase 7a (*grk7a*), G protein-coupled receptor kinase 7b (*grk7b*), and phosphodiesterase 6c (*pde6c*). Notably, *thrb2*^−/−^ larvae exhibited a significant increase in the amount and proportion of first feeding, and their growth rate significantly exceeded that of wild-type controls during the week after feeding. This observation suggests that although the development of the retina may be somewhat affected, *thrb2*^−/−^ larvae show positive changes in feeding behaviour and growth rate, which may be related to their enhanced ability to adapt to their environment. These results provide novel insights into the function of the *thrb2* gene in the visual system and behaviour and may have implications in areas such as fish farming and genetic improvement.

## 1. Introduction

Vision is one of the key sensory functions of vertebrates, playing a pivotal role in their overall life activities [1,2]. The visual system of fish is capable of wavelength discrimination, motion detection, dark vision (low light intensity), and light vision (high light intensity). In order to reduce deaths caused by starvation, the visual system rapidly adapts morphologically and functionally to exogenous nutrient intake in morphology and function after membrane emergence and is able to search and find live bait [3]. However, it should be noted that each species has its own unique visual attributes. Therefore, it is imperative to provide a visual environment for larvae that is conducive to their ability to locate and feed, thereby enhancing their survival and growth prospects when being nurtured. In vertebrates, two types of photoreceptors (cones and rods) express one or more opsins, which are distributed in different patterns across the retina [4]. During the process of development, cones and rods are generated from common retinal progenitor cells (RPCs) [5]. The conversion of light into electrical signals by the cones and rods is then transmitted to the brain’s visual processing centres. Opsin plays an important role in species formation and environmental adaptation in fish. Fish possess opsin genes that determine their visual ability [6,7], and changes in opsin gene expression occur in order to adapt to new environments [8,9]. The majority of fish possess five opsins. Rhodopsin (RH1) is expressed in rod photoreceptors, and four cone opsins are expressed in different cone photoreceptors: short-wavelength-sensitive 1 and 2 (SWS1, SWS2), medium-wavelength-sensitive (RH2), and long-wavelength-sensitive (LWS) photoreceptors [10].

A significant proportion of marine fish larvae, including those of the Asian seabass (*Lates calcarifer*), European hake (*Merluccius merluccius*), and striped bass (*Morone saxatilis*), rely on visual predation of zooplankton as a primary foraging strategy [11,12,13]. It has been demonstrated that zebrafish larvae (*Danio rerio*) are equipped with ultraviolet (UV) vision, which enables them to catch zooplankton in their natural habitat [14]. Meanwhile, in zebrafish, medaka (*Oryzias latipes*), and three-spine stickleback (*Gasterosteus aculeatus*), which feed on zooplankton, UV cones (expressing the UV-Opsin *sws1*) are retained throughout life [14,15,16,17,18]. It has, therefore, been hypothesized that zooplankton is the main determinant driving the retention of UV cones in fish [16,19,20]. UV irradiation increased the feeding rate of first-feeding zooplankton fish, suggesting that UV single cones and SWS1 opsin play a crucial role in their food perception [16,21,22].

Thyroid hormones (THs) are essential for the development and function of the central nervous system, sensory system, respiratory system, and musculoskeletal system [23,24,25,26,27]. In warm-blooded animals, thyroid hormones play an important role in the regulation of energy expenditure [28]. Thyroid hormone receptor beta (*thrb*), a member of the nuclear receptor superfamily, normally acts as a ligand-dependent transcription factor that binds to thyroxine response elements to regulate important physiological and developmental processes by regulating the transcription of target genes in response to ligands. The mechanisms regulating opsin expression in specific cones remain unclear [29,30], but previous in vitro studies have shown that thyroid hormones are associated with cone differentiation [31,32]. Thrb coordinates the cone differentiation of opsin cells in response to the increased thyroxine levels that accompany development [33]. *Thrb1* is expressed in cones, amacrine cells, and ganglion cells, whereas *thrb2* is expressed exclusively in cones [34,35,36]. Despite the detection of *thrb1* in cones, *thrb1* knockout mice exhibited only minor changes in opsin photopigment expression, unremarkable electroretinographic responses, and no notable changes in cones [36].

In this study, we constructed a *thrb2* medaka mutant using CRISPR/Cas9 technology with the objective of investigating the effect of *thrb2* on retinal development and first feeding in fish. This study represents the first analysis of the combined effects of *thrb2* and *sws1* on the first feeding and growth of fish, providing a theoretical foundation for addressing the challenge of first feeding in larvae.

## 2. Materials and Methods

### 2.1. Animals

The Japanese medaka (*Oryzias latipes*) were reared in a recirculating water system at the Mandarin Research Centre of Huazhong Agricultural University (Wuhan, China). The room temperature was maintained at 26–28 °C throughout the year, with a cycle of 14 hours of (h) light and 10 h of darkness per day. The fish were fed twice a day with *Artemia salina*. The embryos were placed in a medaka embryo medium (MEM) [37] and incubated at 28 °C in an incubator.

### 2.2. Mutants

The DNA sequence of medaka *thrb2* was retrieved from the National Center for Biotechnology Information (NCBI) database (https://www.ncbi.nlm.nih.gov/), and the single guide RNA (sgRNA) was designed on the first exon using the online software CCTop https://cctop.cos.uni-heidelberg.de:8043/help.html (accessed on 20 February 2025). The sequences of the sgRNA primers and detection primers are shown in Table S1. PMD-19T was used as a template for PCR amplification. The amplification products were purified using the DNA Gel Extraction kit (Simgen, Hangzhou, China), and the purified products were transcribed in vitro using the TranscriptAid T7 High Yield Transcription kit (Thermo, Scientific, San Diego, CA, USA). In vitro transcript samples were recovered by precipitation using LiCl. Thrb2-sgRNA (100 ng/µL) and Cas9 proteins (NEB, Ipswich, MA, USA) were mixed together and co-injected into 1–2-cell stage medaka embryos. Approximately 10 medaka embryos were randomly selected 24 h after injection, the genomic DNA of medaka embryos was rapidly extracted with the kit (FOREGENE, Chengdu, China), and the mutation was detected by PCR amplification. The F0 mutation was cultured into adult fish and then sequenced by clipping the tail fin. The mutant F0 was paired with the wild type (WT) to produce F1 individuals, and the homozygous F2 was obtained by pairing F1 generations with the same type of knockout mutation.

### 2.3. First-Feeding Analysis of Larvae

The yolk sac was almost entirely consumed at 6 days post-hatching (dph) in medaka; therefore, WT and *thrb2*^−/−^ medaka at 6 dph were selected for the initial evaluation of first feeding. The measurement of first feeding in medaka was based on a previously reported method with some modifications [38]. In an 8 × 8 × 6 cm transparent box, 200 mL of MEM was added, 30 medaka larvae were placed for 30 minutes (min) to acclimatize, and then fed with *A. salina* (approximately 1000). Larvae were anesthetized with tricaine methanesulfonate (MS-222) after 30 min of feeding and fixed overnight with 4% paraformaldehyde (PFA) for feeding analysis. The experiment was repeated three times. The orange area of the *A. salina* in the digestive tract was photographed with a stereomicroscope (Leica, GER) and measured with ImageJ 1 software (IJ 1.46r).

### 2.4. Growth and Survival Analyses

A total of 120 fish of each medaka genotype were randomly selected and placed in eight transparent culture boxes, with each box containing 30 fish and 200 mL of water. Medaka were fed with *A. salina* once a day, and the water was changed after 1 h to ensure a consistent daily feeding time. The survival rate of medaka larvae in each group was counted at 1, 3, 5, and 7 days (d) (*n* = 100). After overnight fixation with 4% PFA, photographs were taken with a stereomicroscope, and the total length was measured. The experiment was repeated three times.

### 2.5. RNA Isolation and Quantitative RT-PCR

Eighteen 6 dph larvae and 3-month-old adults were randomly selected separately, and their eyes were removed after being anesthetized with MS-222. The eyes of every third fish were mixed together. The total RNA was extracted from the samples using TRIzol Reagent (Takara, Tokyo, Japan), and cDNA synthesis was performed using the Reverse Transcription Kit (Vazyme, Nanjing, China), which was then stored at −20 °C. Quantitative Real-Time PCR (qRT-PCR) was used to detect gene expression, and *β-actin* was selected as the reference gene. The primers involved were designed using the Primer Premier 6 software, as shown in Table S1. A total of 10 μL of the qRT-PCR reaction system was used, consisting of a 5 μL SYBR mixture (Vazyme, Nanjing, China), 3.6 μL ddH_2_O, 0.2 μL each of upstream and downstream primers, and 1 μL cDNA. The qRT-PCR program consisted of pre-denaturation at 95 °C for 30 seconds (s), 40 cycles of denaturation at 95 °C for 10 s and annealing at 58 °C for 30 s, and melting curves from 65 °C to 95 °C with a gradual increase of 0.5 °C s-1, with data collected every 6 s.

### 2.6. Hematoxylin–Eosin (HE) Staining

Medaka larvae at 6 dph (*n* = 6) were randomly selected, and fresh eye tissues were removed and then rapidly fixed in an FAS eye fixative (Servicebio, Wuhan, China) for more than 24 h. After fixation, paraffin sections were taken to observe the development of the retina in medaka eyes using Hematoxylin–Eosin (HE) staining, and the retinal structure was measured using the Case-Viewer software (C.V.2.6).

### 2.7. Movement Behaviour Analysis

The swimming speed of larvae was determined using the DanioVision^TM^ behavioural monitoring system (Noldus, The Netherlands) with the accompanying EthoVision^®^ XT 14 video tracking software (EthoVision XT 14). Medaka larvae were placed in 24-well plates, and a 1 mL culture medium was maintained in each well. The larvae used for testing should be normally developed and free of any malformations. For light/dark behaviour analysis [39], prior to the commencement of the testing process, medaka larvae to be tested in 24-well plates were allowed to acclimate to 28 °C for 10 min. The software was set to a continuous alternating light/dark cycle (3 min light–3 min dark–3 min light). In the video-tracking system, the movement speed (cm/s) of medaka larvae was collected every minute. For light response analysis [40], before testing, medaka larvae were acclimatized in the dark for 30 min. After light stimulation for 2 min following the end of dark adaptation, swimming speed was counted for the last 2 min of dark stimulation and the first 2 min of light stimulation. Mean swimming speed was collected every 2 min in the dark and every 10 s in the light.

### 2.8. Statistical Analysis

All values were analyzed using SPSS Statistics 24.0 software and expressed as mean ± standard error (mean ± SEM). The normality of the data was first tested by the Shapiro–Wilk test. Values for each group were subjected to one-sample *t*-tests, and independent samples *t*-tests were used to compare differences between groups. *p* < 0.05 indicates a significant difference marked with “*”; *p* < 0.01 indicates a highly significant difference marked with “**”.

## 3. Results

### 3.1. Construction of thrb2 Mutant in Medaka

**Figure 1:**
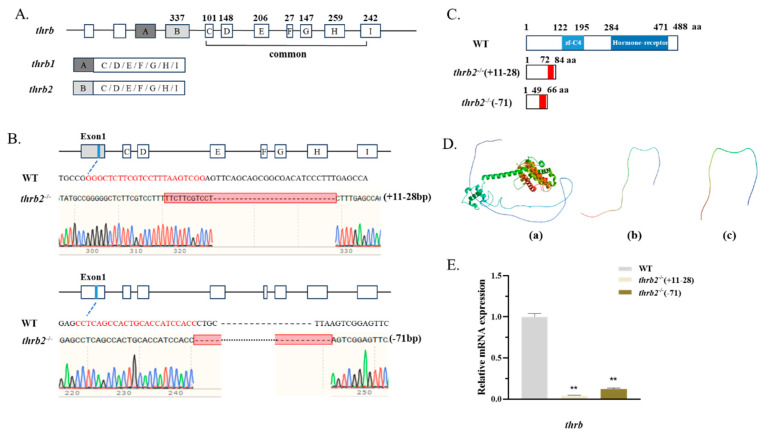
The genetic structure and characterization of the *thrb2* mutant. (**A**) Gene structure of *thrb2*. (**B**) The location of mutations in *thrb2* knockouts. The red labels indicate sgRNAs, and the red boxes indicate the locations of mutations. (**C**) THRB2-predicted domain and amino acid translation termination sites in knockouts. The red colour represents sequences with incorrectly expressed amino acids. (**D**) Prediction of the tertiary structure of THRB2 protein. (a) WT, (b) *thrb2*^−/−^ (+11–28), and (c) *thrb2*^−/−^ (−71). (**E**) Expression of *thrb2* in the mutants. The results are shown as mean *±* SEM. *p* < 0.01 indicates a highly significant difference marked with “**”; and *n =* 6 for each genotype.

*Thrb* has two splice variants, *thrb1* and *thrb2*, with seven shared exons (Figure 1A). The predicted domains found that zf-C4 and hormone-receptor domains were located at a common exon location (Figure 1C). The sgRNA was designed on *thrb2*-specific exons, and two mutants of *thrb2* were obtained by CRISPR/Cas9 (Figure 1B). The *thrb2*^−/−^ (+11-28) homozygote has protein translation termination at the 84th amino acid position, and the *thrb2*^−/−^ (−71) homozygote has translation termination at the 66th amino acid position. The predicted tertiary protein structure of *thrb2* also confirmed the knockdown results (Figure 1D). Compared with the wild-type group, the *thrb2* mRNA expression level in the *thrb2*^−/−^ (+11-28) and *thrb2*^−/−^ (−71) mutants were significantly decreased (Figure 1E).

### 3.2. Analysis of Opsin Expression After thrb2 Knockout

**Figure 2:**
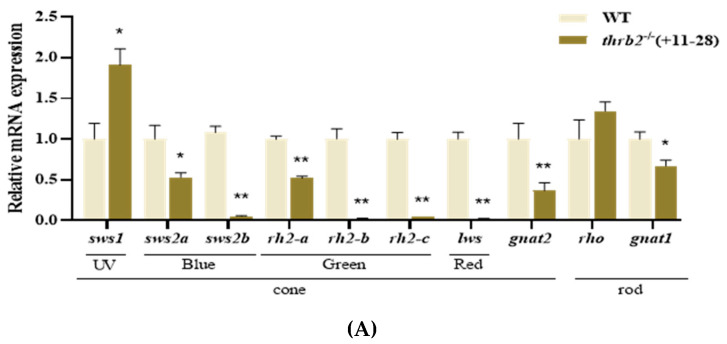

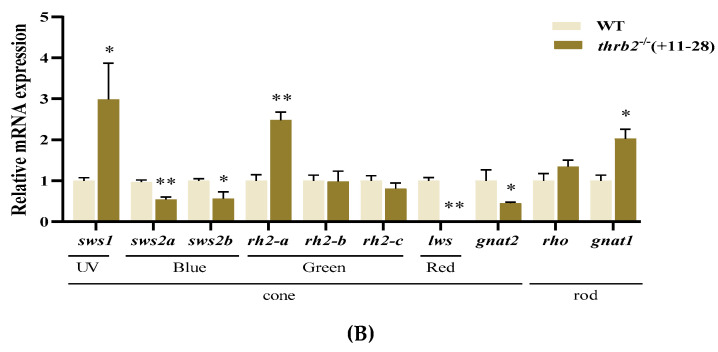
The expression of opsins in wild-type and *thrb2*^−/−^ (+11-28) medaka at 6 dph (**A**) and 3 months old (**B**). Relative expression levels are presented as mean *±* SEM. *p* < 0.05 indicates a significant difference marked with “*”, *p* < 0.01 indicates a highly significant difference marked with “**”, and *n* 6.

RT-PCR analyses revealed that the expression of *sws1* was significantly increased, while short-wave-sensitive 2a (*sws2a*) was significantly decreased in medaka larvae at 6 dph (*p* < 0.05) (Figure 2A). The expression of all other opsins in visual cones showed highly significant reductions (*p* < 0.01), whereas visual rods showed no statistically significant differences in rho expression (*p* > 0.05), and G protein subunit alpha transducin 1 (*gnat1*) expression was significantly reduced (*p* < 0.05). The expression of *sws1* was significantly elevated (*p* < 0.05), whereas the expression of *sws2a* and short-wave-sensitive 2b (*sws2b*) was significantly reduced in 3-month-old medaka single cones (*p* < 0.05) (Figure 2B). The expression of rhodopsin 2a (*rh2-a*) in double cones was highly significantly increased (*p* < 0.01), the expression of long-wave-sensitive (*lws*) opsin was highly significantly decreased (*p* < 0.01), and the expression of *gnat1* in rods was highly significantly increased (*p* < 0.01). This finding suggests that the *thrb2* knockout may have specific effects on the expression of different types of opsins in the retina.

### 3.3. Analysis of the Effects of Retinal Development

**Figure 3:**
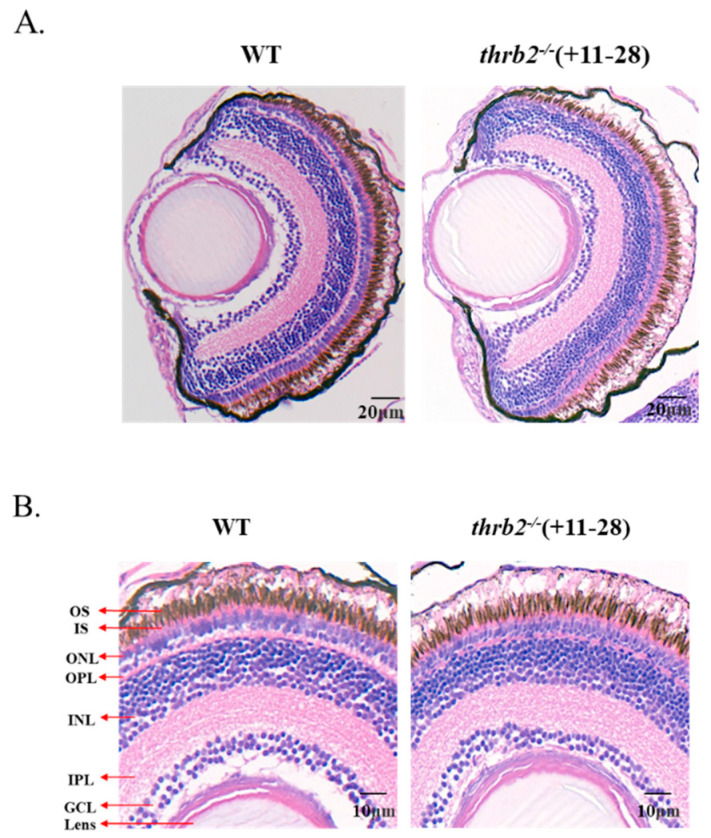

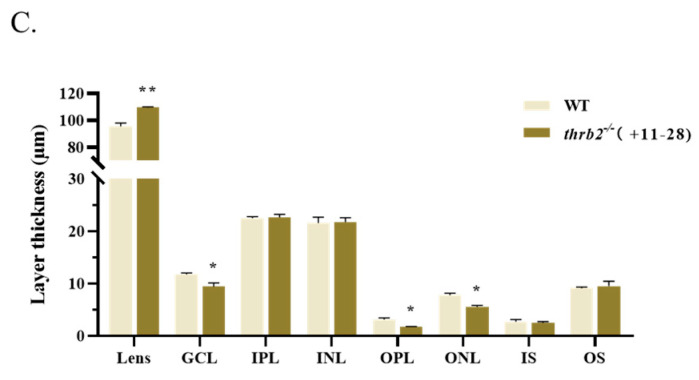
Effects of *thrb2*^−/−^ (+11-28) medaka on retinal stratification and photoreceptor development compared to wild type. (**A**,**B**) Histological and morphological analysis of the 6 dph medaka retina by HE staining. GCL, ganglion cell layer; IPL, inner plexiform layer; INL, inner nuclear layer; OPL, outer plexiform layer; ONL, outer nuclear layer; IS, inner segment; OS, outer segment. Scale bar: 20 μm, 10 µm. (**C**) Quantitative analysis of the thickness of retinal cell stratification. All data are expressed as mean *±* SEM. “*” indicates *p* < 0.05, “**” indicates *p* < 0.01, and *n =* 6 for each genotype.

To investigate the effects of *thrb2* knockout on retinal stratification and photoreceptor development in medaka, 6 dph wild-type and knockout medaka were subjected to paraffin sectioning and HE staining. As illustrated in Figure 3A-B, *thrb2*^−/−^ (+11-28) medaka had normal retinal morphology and obvious cell layer stratification. However, the thickness of these cell layers was found to be altered. There was a significant increase in lens thickness (*p* < 0.01) and a significant decrease in ganglion cell layer (GCL), outer plexiform layer (OPL), and outer nuclear layer (ONL) thickness in the *thrb2*^−/−^ (+11-28) medaka (*p* < 0.05), with no significant difference in the thickness of the other cell layers (*p* > 0.05) (Figure 3C). In comparison to the wild type, the outer segments of photoreceptor cells in the *thrb2*^−/−^ (+11-28) medaka exhibited partial hollowness and poor connectivity with the retinal pigment epithelial (RPE), the GCL was closer to the lens, and the cells in ONL demonstrated a more compact arrangement (Figure 3A,B); these changes suggest that retinal development may be affected.

### 3.4. First-Feeding Test

**Figure 4:**
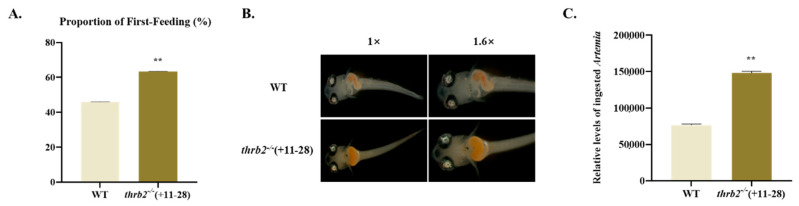
Assessment of first-feeding capacity in wild-type and *thrb2*^−/−^ (+11-28) medaka. (**A**) Proportion of first feeding in wild-type and *thrb2*^−/−^ (+11-28) medaka. (**B**) Images were photographed 30 min after ingestion. (**C**) Quantitative analysis of food intake at first feeding. The values shown are mean *±* SEM. “**” indicates *p* < 0.01.

In order to evaluate the first-feeding capacity of the *thrb2*^−/−^ (+11-28) medaka, a quantitative analysis was conducted of the proportion of first feeding and food intake by medaka larvae at 6 dph. The proportion of first feeding in the *thrb2*^−/−^ (+11-28) medaka was significantly higher compared to the wild-type medaka (*p* < 0.01) (Figure 4A). The results of stereomicroscopic observations indicated that the contents of the digestive tract of the *thrb2*^−/−^ (+11-28) medaka were markedly larger than those of the wild-type medaka. Following analysis using ImageJ 1 software, it was evident that the food intake of the *thrb2*^−/−^ (+11-28) medaka was significantly higher than that of the wild-type medaka (*p* < 0.01) (Figure 4B,C).

### 3.5. Growth and Survival Analysis

**Figure 5:**
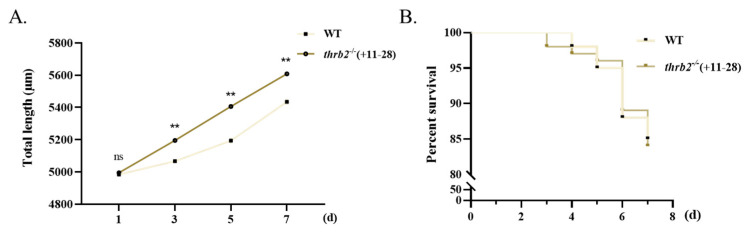
Effect of *thrb2* on medaka growth (**A**) (*n* = 30/group) and survival (**B**) (*n* = 100/group) compared to wild type within seven days of first feeding. d, day; error bars, mean ± SEM. *p* < 0.01 indicates a highly significant difference marked with “**”.

The impact of *thrb2*^−/−^ (+11-28) on the growth and survival of larvae following first feeding was also investigated. It was found that the body length of the *thrb2*^−/−^ (+11-28) medaka was significantly longer than that of the wild-type medaka at 3 d, 5 d, and 7 d after first feeding (*p* < 0.01) (Figure 5A). The *thrb2*^−/−^ (+11-28) medaka had no significant impact on survival during the seven-day feeding period (*p* > 0.05) (Figure 5B).

### 3.6. Locomotor Behaviour of Larvae

**Figure 6:**
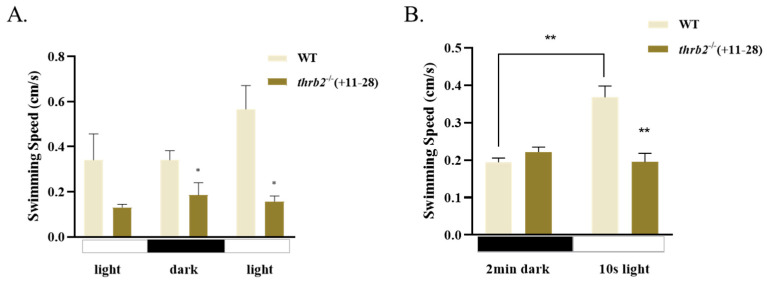
Swimming speed of 6 dph wild-type and *thrb2*^−/−^ (+11-28) medaka in the light/dark behavioural assays (**A**) and light-response assays (**B**). Compared with the wild-type medaka, “*” indicates *p* < 0.05 and “**” indicates *p* < 0.01; all data are expressed as mean *±* SEM (*n* = 24).

To examine alterations in swimming capability in the *thrb2* mutant, we performed light/dark behavioural assays and light-response assays on 6 dph wild-type and *thrb2*^−/−^ (+11-28) medaka. The results of the light/dark behavioural assays indicated that the swimming speed of the *thrb2*^−/−^ (+11-28) medaka was significantly lower than that of the wild type during light/dark/light alternation (*p* < 0.05) (Figure 6A). In light-response assays, there was no significant difference in swimming speed between wild-type and *thrb2*^−/−^ (+11-28) medaka in the dark (*p* > 0.05) (Figure 6B). However, the swimming speed of the wild-type medaka was significantly higher than that of the *thrb2* mutant in 10 s light stimulation (*p* < 0.05). The swimming speed of the wild-type medaka was significantly higher in the light than in the dark conditions (*p* < 0.05). In contrast, the *thrb2*^−/−^ (+11-28) medaka exhibited no significant difference in swimming speed between the light and dark conditions (*p* > 0.05).

### 3.7. Expression Analysis of Genes Related to Phototransduction Process

**Figure 7:**
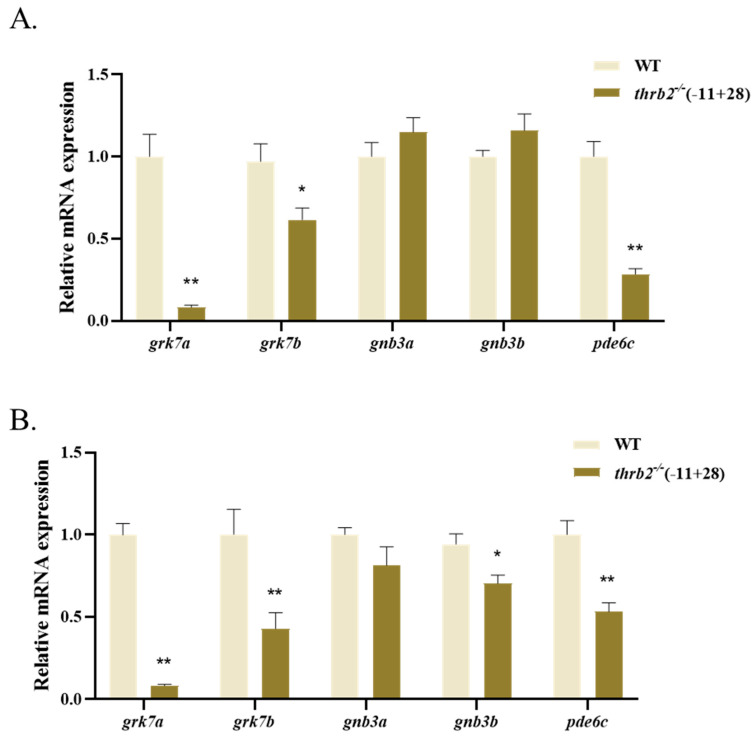
The expression of genes involved in the phototransduction process in wild-type and thrb2^−/−^ (+11-28) medaka at 6 dph (**A**) and 3 months old (**B**). Relative expression levels are presented as mean ± SEM. *p* < 0.05 indicates a significant difference marked with “*”, *p* < 0.01 indicates a highly significant difference marked with “**”, and *n* = 6.

The expression of genes involved in the phototransduction process was quantified at 6 dph and in 3-month-old medaka (Figure 7). Expressions of G protein-coupled receptor kinase 7a (*grk7a*), G protein-coupled receptor kinase 7b (*grk7b*), and phosphodiesterase 6c (*pde6c*) were significantly reduced at 6dph and in 3-month-old medaka compared to the wild type (*p* < 0.05), but guanine nucleotide-binding protein (G protein), beta polypeptide 3a (*gnb3a*) showed no significant difference (*p* > 0.05). No notable discrepancy was observed in guanine nucleotide-binding protein (G protein) and beta polypeptide 3b (*gnb3b*) at 6 dph (*p* > 0.05); however, the expression of *gnb3b* was markedly diminished in 3-month-old medaka (*p* < 0.05).

## 4. Discussion

The regulation of long-wavelength vision in vertebrates is dependent upon thyroid hormone (TH) signalling [27]. *Thrb2* is a TH-activated transcription factor expressed in immature cones that regulates cone differentiation and opsin expression and is required for the expression of long-wavelength-sensitive opsin (*lws*) in red cone photoreceptors [27,41]. In this study, two mutants of *thrb2* were constructed. Despite the lack of validation of the expression level of *thrb2* at the protein level (due to the unavailability of an antibody specific to *thrb2*), a notable reduction in the expression of *thrb2* at the mRNA level was observed, indicating the efficacy of the knockout result.

Our study showed that the expression of *sws1* was significantly increased in 6 dph larvae and 3-month-old adults of *thrb2*^−/−^ medaka, and conversely, the expression of blue opsins (*sws2a*, *sws2b*) and red opsins (*lws*) were all significantly lower. Consistent with our findings, in human retinas lacking *thrb*, cone development is characterized by the formation of single cones [42]. In the absence of the *thrb* gene in mice, red double cones are converted to UV single cones [27], and the expression of *Opn1mw* is reduced, whereas the expression of *Opn1sw* (the zebrafish homologue of *sws1*) is increased [41,43]. The retina of teleost eyes is usually composed of several functional layers [44], and down-regulated expression levels of opsin genes are indicative of potential retinal photoreceptor damage. Opsin deficiency has been demonstrated to have a detrimental effect on the stability of photoreceptor cell membranes, which can ultimately result in cell death [45]. Further observation of the retinal structure revealed that the thickness of the *thrb2*^−/−^ lens was significantly increased, and the thickness of the GCL, OPL, and ONL were significantly reduced. The GCL was observed to be in closer proximity to the lens, and the cells of the ONL were found to be more closely arranged. Thus, *thrb2* may contribute to retinal developmental defects by altering retinal structure and opsin gene expression levels.

In most fish larvae, vision plays a very important role in the first feeding because of the rapid transmission of signals to the brain, and the opsin genes a fish possesses determine its visual ability [6,7]. In the context of larval feeding on zooplankton, *sws1* is typically characterized by elevated levels of expression. Consequently, the provision of a visual environment conducive to larval discovery and feeding is imperative to enhance the survival and growth rates of larvae when cultivated. Treatment of trout larvae with thyroxine reduced the sensitivity of larvae to zooplankton and accelerated the conversion of UV cones to S cones in the retina, which enhanced the ability of trout larvae to forage for zooplankton [16]. In comparison to the wild-type zebrafish, the mutants with a reduced number of UV cones exhibited a mean distance and angle of feeding towards zooplankton that were 24% and 90% smaller, respectively [22]. It was observed that there was a notable increase in the proportion and food intake of *thrb2* purists during the first-feeding period and that growth was significantly accelerated following the deletion of *thrb2*. A significant reduction in both food intake and growth was observed in *sws1*^−/−^ medaka larvae [46]. The findings indicate that *thrb2* modulates the expression of *sws1* in visual development, which subsequently influences the processes of the first feeding and the growth and development of larvae. This observation suggests that although the development of the retina may be somewhat affected, *thrb2*^−/−^ larvae show positive changes in feeding behaviour and growth rate, which may be related to their enhanced ability to adapt to their environment.

It is evident that a considerable number of fish behaviours are contingent on their visual function [47]. Consequently, alterations in visual function and retinal structure can affect visually guided motor behaviour [48]. Light adaptation enables the vertebrate visual system to operate under a wide range of ambient illumination. Phototransduction regulation is considered to be the main mechanism of light adaptation [49]. Rods and cones respond to light and transmit signals to bipolar cells [50]. Cones mediate vision and colour vision under bright light conditions, whereas rods mediate vision under dim light conditions [51]. The function of cones is to detect light, whereas rods are responsible for detecting dark. In both light/dark behavioural assays and light-response assays, the speed of wild-type medaka was significantly higher than that of the *thrb2* knockout group after light exposure. As Min Wei et al. assert, TH signalling performs a pivotal function in the coordination of Muller glial cells (MGs) and other retinal cells, thereby regulating light adaptation. Light exposure has been demonstrated to elevate *Dio2* in MGs, while *thrb2* exhibits high levels of expression in cones, thus enabling adaptation to the prevailing light environment [17]. The quantification of genes involved in cone light transduction in medaka at 6 d and 3 months revealed decreased expression of *grk7a*, *grk7b*, and *pde6c*. It was hypothesized that the photoconduction mechanism would be impaired in the *thrb2*^−/−^ larvae, thus further reducing their swimming speed.

## 5. Conclusions

In summary, a knockout mutant of *thrb2* was constructed, revealing that this gene plays an important role in retinal development and first feeding. The results demonstrated that the absence of *thrb2* had a substantial impact on opsin gene expression and cell stratification within the retina. Furthermore, the study established that *thrb2* results in reduced swimming speed in larvae under light by affecting the expression of cone light transduction genes. A particularly noteworthy finding emerged from the study: the *thrb2* knockout led to a substantial augmentation in the amount and proportion of first feeding in larvae. The model provides novel methodologies and concepts for investigating the means by which larvae can augment their intake of zooplankton and feeding proportion.

## Data Availability

The original contributions presented in this study are included in the article/Supplementary Materials. Further inquiries can be directed to the corresponding author(s).

## Supplementary Materials

The following supporting information can be downloaded at https://www.mdpi.com/article/10.3390/cells14050386/s1. Table S1: Primers used in the experiment.
