# Supplementary material for: The Beneficial Role of the Thyroid Hormone Receptor Beta 2 (thrb2) in Facilitating the First Feeding and Subsequent Growth in Medaka as Fish Larval Model
Source: Cells. 2025 Mar 6;14(5):386. doi: 10.3390/cells14050386 (PMC11898640; doi:10.3390/cells14050386)
Supplement: Supplementary file 1 [file cells-14-00386-s001.zip › cells-3452615-supplementary.pdf]

Table. S1 Primers used in the experiment

Gene name	Primer name	sequences (5′-3′)	Annealing temp (℃)
	thrb2-sg1-F	GTAATACGACTCACTATAGGGCTCTTCGTCCTTTAAGTGTTTTAGAGCTAGAAATAGC	62
	thrb2-sg-R	AAAAGCACCGACTCGGTGCC	
	thrb2-sg3-F	GTAATACGACTCACTATAGGTGGATGGTGCAGTGGCTGGTTTTAGAGCTAGAAATAGC	
·*thrb2*	*thrb2*-C-F	GGCTCAACCTCATCAAGT	56
	thrb2-C-R	GCATCTCCATCAATCAATCA	
	thrb2-RT-F	TTGAGCCACTTCCACTAC	58
	thrb2-RT-R	CCAGGTAACTCGGTATGTAA	
sws1	*sws1*-RT-F	ACAAAGACTACCGGCTCGTCA	58
	sws1-RT-R	GCTTCCTCCATCTTCTTCCGA	
sws2a	*sws2a*-RT-F	CCACCTGAACTACATCCTG	58
	sws2a-RT-R	CACAGCAAGAGACCACAG	
sws2b	*sws2b*-RT-F	TTCTGCTTTGCCGTCCCTT	58
	sws2b-RT-R	AAGCAACAACCATGACGACCA	
rh-2a	*rh-2a*-RT-F	TTTGGTTGGCTGGTCAAGGT	58
	rh-2a-RT-R	CACAAGGCTGCCATAGGTGA	
rh-2b	*rh-2b*-RT-F	TGCACTTCTTCGTCCCAGTC	58
	rh-2b-RT-R	CCAAGCTACCAGGAAGCCAA	
rh-2c	*rh-2c*-RT-F	CTTCTGTGCCGTTGAGGGAT	58
	rh-2c-RT-R	ACAAGCCAGAGCCATTACCC	
lws	*lws*-RT-F	TGAGGGCTATGTGGTCTCCA	58
	lws-RT-R	ACCTGCTCCATCCAAAGACG	
rho	*rho*-RT-F	CTCGTTACATCCCAGAGGGC	58
	rho-RT-R	ATGATGACAACCATGCGGGT	
gnat1	*gnat1*-RT-F	AAGCCCATCTGAGCATGTGT	58
	gnat1-RT-R	TGATGATGTCGGTTACGGCA	
gnat2	*gnat2*-RT-F	GTGAGTCGGGAAAAAGCACC	58
	gnat2-RT-R	CCAGAGCCAGAACCGAAGTC	
grk7a	*grk7a*-RT-F	CCACCAAGGACGTCATCCA	58
	grk7a-RT-R	CGTGTTTCCTCGGATCCTC	
grk7b	*grk7b*-RT-F	TGAGAGGCAGAAGATTACTGACA	58
	grk7b-RT-R	GCACAACACAAACCTCACCAAA	
gnb3a	*gnb3a*-RT-F	CAGCCTCAAAGACCAGATTACGG	58
	gnb3a-RT-R	TCATCTGCACACGTCCCAC	
gnb3b	*gnb3b*-RT-F	GAAAGCTGAAATGGATGCACTGAA	58
	gnb3b-RT-R	ACATGCTCCCATCATTTGCTG	
pde6c	*pde6c*-RT-F	CGGCAAACTCCAAAAAGCCA	58
	pde6c-RT-R	TGAGGGGGAAGGTGAGCATA	
β-actin	*β-actin*-RT-F	TATCATTCGCCTGAAACCGAT	58
	β-actin-RT-R	CTTTGCACATGCCAGATCCG	
